# Synthesis and Characterization of “Ravine-Like” BCN Compounds with High Capacitance

**DOI:** 10.3390/ma11020209

**Published:** 2018-01-29

**Authors:** Dongping Chen, Yanzhen Huang, Xinling Hu, Rongkai Li, Yingjiang Qian, Dongxu Li

**Affiliations:** College of Materials Science and Engineering, Huaqiao University, Xiamen 361021, China; 1511302040@hqu.edu.cn (D.C.); 15737567342@163.com (Y.H.); 18224566342@163.com (X.H.); liyungkai@yeah.net (R.L.); qian1358_love@163.com (Y.Q.)

**Keywords:** pyrolysis, boron carbonitride, electrochemical performance, supercapacitors

## Abstract

A series of “ravine-like” boron carbonitrides (abbreviation: BCN) were synthesized by a green precursor pyrolysis method at different temperatures (about 700–1100 °C). The highest electrochemical performance of BCN-800 (Named BCN-temperature) electrode was observed, because the “ravine-like” structure can significantly increase the contact area and improve the wettability between electrode and electrolyte. The BCN electrode exhibited ultrahigh specific capacitance 805.9 F/g (at a current density of 0.2 A/g), excellent rate capability, and good cycling stability (91%) after 3000 cycles at a current density of 8 A/g, showing high potential applications in supercapacitors.

## 1. Introduction

In recent years, two-dimensional (2D) materials have attracted considerable attention because of their unique physical and chemical properties, which ensure excellent performance in many fields, such as catalysts, semiconductors, energy storage, and electronics [1,2,3]. Among these 2D materials, boron carbonitride (BCN) has attracted the interest of physical, chemical, and materials science researchers. Numerous theoretical and experimental studies have shown that BCN compounds exhibited various potential applications because of their excellent electrical, optical, thermal, and mechanical properties, which are attributed to their similar structures to BN and C and their adjustable physical and chemical properties [4,5,6]. At present, most of the BCN prepared in experiments belong to C-rich BCN compounds. Moreover, many preparation methods were presented, including magnetron sputtering, chemical vapor deposition, physical vapor deposition, and high-temperature and high-pressure (HTHP) methods. Liu et al. [7] successfully used the HTHP method to synthesize nearly-transparent BCN diamonds. Ma et al. [8] used the method of “thermal substitution” to dope C into BN to prepare BCN nanosheets which had a controllable band-gap and exhibited excellent nonlinear optical performance. Karbhal et al. [9] used the thermal decomposition method to synthesize BCN nanosheets with a high specific capacitance (244 F/g). Finding and exploring new domains of applications of BCN compounds are hot spots in the field of materials science research.

Energy, the material basis of human activities, has been the focus of attention around the world. Supercapacitors exhibit excellent performance with high specific capacity, high energy density, high power, and long life cycle [10]. Thus, electric cars, mobile communications, defense science and technology, and many other fields have broad prospects (i.e., real green energy) [11]. In recent years, research on the use of BCN materials in supercapacitors has often been reported. Research shows that boron and nitrogen co-doping in carbon material effectively changes the electron donor/acceptor characteristics, which improves the capacitance performance of carbon materials [12].

This work presented a pyrolysis method to produce “ravine-like” BCN compounds at different temperatures. A certain amount of boric acid was mixed with 2,4,6-tri(2-pyridyl)-1,3,5-triazine to form the precursor, and the precursor was pyrolyzed to prepare BCN compounds in nitrogen atmosphere. Capacitance performance tests show that the specific capacitance of the “ravine-like” BCN compounds was 2–3 times higher than that reported in the literature under the same test conditions, which exhibits ultrahigh specific capacitance and excellent rate capability and has potential applications in supercapacitors.

## 2. Results and Discussion

**Figure 1:**
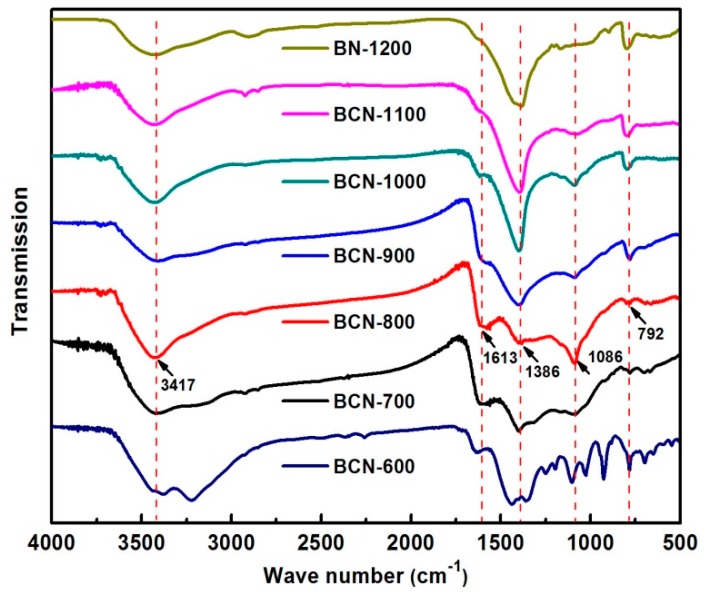
Fourier transform infrared (FTIR) spectra of boron carbonitride (BCN)-600, BCN-700, BCN-800, BCN-900, BCN-1000, BCN-1100, and BN-1200.

The Fourier transform infrared (FTIR) spectra of BCN samples were collected to investigate the common features of as-prepared BCN samples under different temperatures (Figure 1). The absorption peaks at 792 and 1386 cm^−1^ are attributed to the bending vibration of out-of-plane B–N–B and transverse stretching vibration of in-plane B–N bonds in all samples [13,14]. The small absorption peaks at 1086 and 1613 cm^−1^ correspond to the B–C vibrations and *sp*^2^ C–N bonds [15,16]. Another broad peak at 3417 cm^−1^ is attributed to the O–H stretching vibration mode [17]. Regularly, intensities of B–N and B–N–B peak increased with increasing pyrolysis temperature, whereas the B–C and C–N vibration modes decreased sharply. Absorption peaks of precursor appeared at 600 °C, meaning that the precursor is still included. The FTIR results confirm the formation of atomic-level ternary BCN hybrid structures.

**Figure 2:**
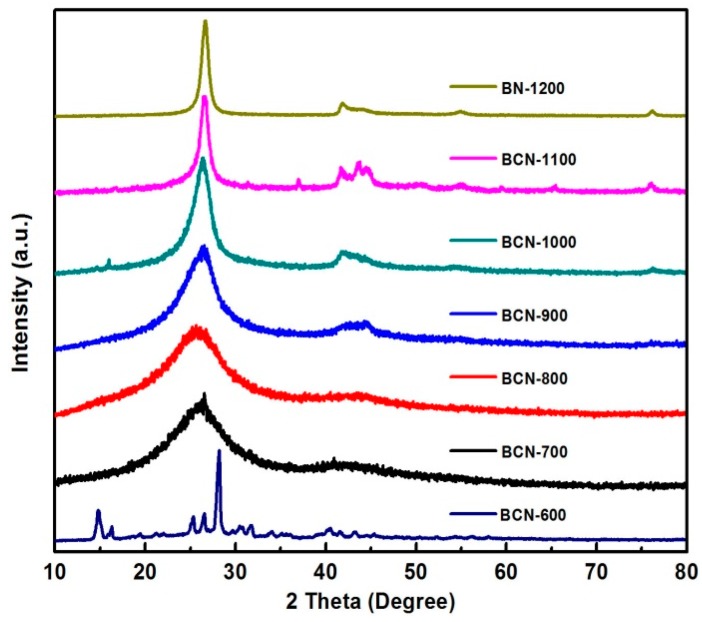
XRD pattern of BCN-600, BCN-700, BCN-800, BCN-900, BCN-1000, BCN-1100, and BN-1200.

Figure 2 shows the XRD patterns of BCN samples obtained at different pyrolysis temperatures, which have two main broad reflections centered at 26.2° and 43.6°, attributed to the (002) and (100) planes, respectively [18,19]. Furthermore, the XRD patterns at relatively low pyrolysis temperatures (700 °C to 900 °C) are significantly broader than that of the other samples, which indicates the presence of the amorphous phase, composed of typical graphite-like BCN materials. With increase of pyrolysis temperature, the diffraction peak became sharper, indicating better crystallization. At 1100 °C, the product began to phase separate and BN was included mainly at 1200 °C. Obviously, unreacted precursor existed in BCN-600, in good agreement with FTIR results. Additionally, phase separation was observed over 1200 °C.

**Figure 3:**
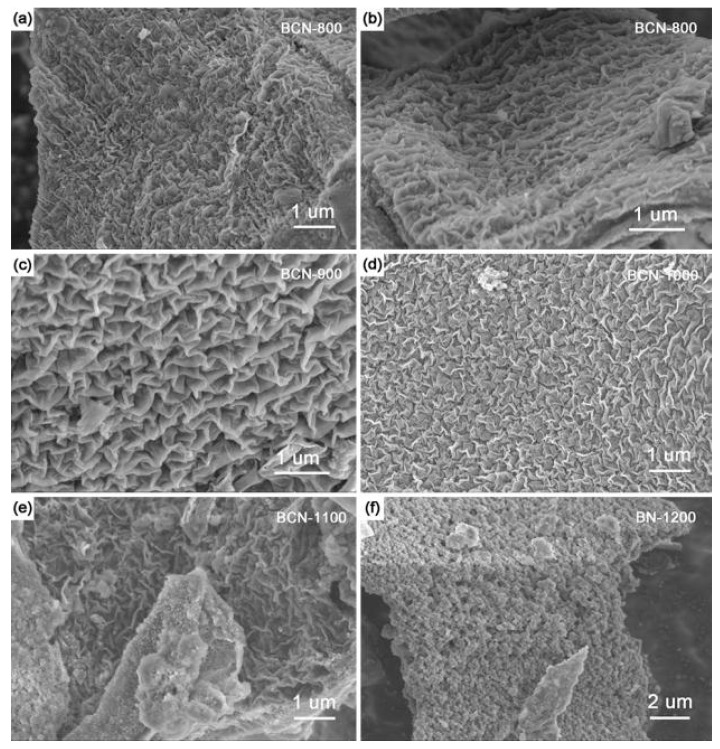
SEM images of (**a**,**b**) BCN-800; (**c**) BCN-900; (**d**) BCN-1000; (**e**) BCN-1100; (**f**) BN-1200.

**Figure 4:**
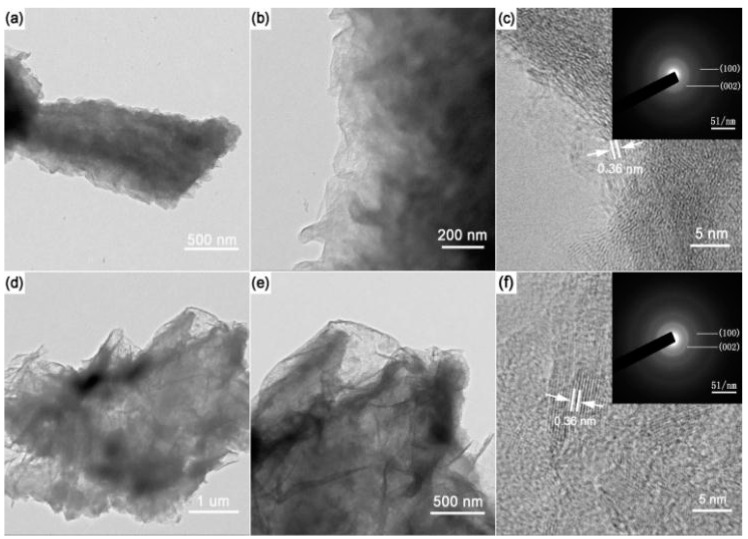
(**a**–**c**) TEM images and the corresponding HRTEM images of BCN-800; (**d**–**f**) TEM images and the corresponding HRTEM images of BCN-900.

SEM was employed to investigate the morphology of the samples. As shown in Figure 3, “ravine-like” BCN compounds can be observed, with sizes of 150 to 200 nm. However, the morphology of the “ravine-like” BCN was destroyed as temperature increased, and basically disappeared over 1200 °C. TEM and corresponding HRTEM images were collected to analyze the microstructure of samples. The “ravine-like” structures of BCN-800 and BCN-900 were presented in HRTEM images (Figure 4). The lattice spacing was calculated to be 0.36 nm, matching the (002) plane of samples in XRD. Meanwhile, with the increase of temperature, the crystal density of the samples increased and the crystal became larger (as seen in the Supplementary Figure S1A(d)). These conclusions are consistent with the results of XRD characterization. In addition, with the increase in pyrolysis temperature, the “ravine-like” morphology was obviously reduced, which might be attributed to disordered crystallization filling in the ravine.

**Figure 5:**
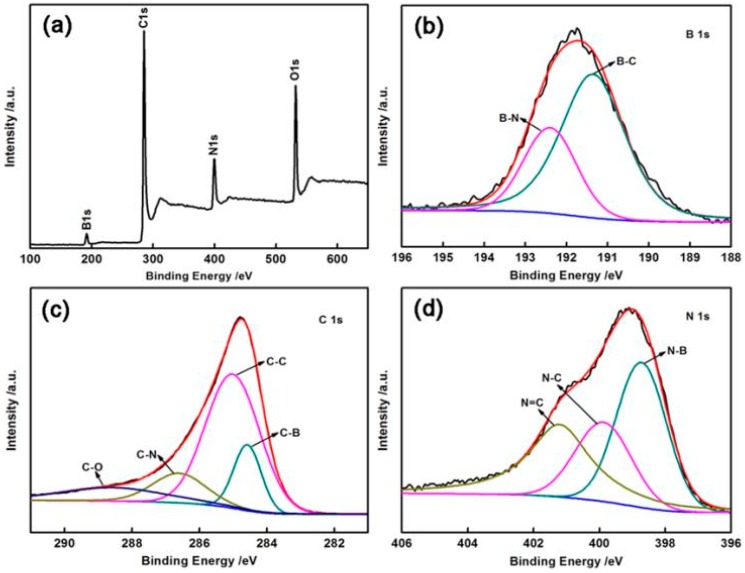
(**a**) The survey scan of X-ray photoelectron spectroscopy (XPS) on BCN-800; (**b**) B 1s XPS peak; (**c**) C 1s XPS peak and (**d**) N 1s XPS peak.

For further characterization of the elemental composition, X-ray photoelectron spectroscopy (XPS) experiments were performed (Table 1 and Figure 5). The elemental compositions of BCN-800 were calculated to be 10.15, 62.66, 12.17, and 15.02 at % for B, C, N, and O, respectively. With the increase of temperature, the contents of B and N increased. When the temperature increased to 1200 °C, boron nitride (BN) was the main composition, which is consistent with the above results. In addition, the O 1s signal could be ascribed to moisture and other gases adsorbed on the surface because of the strongly oxophilic nature of B in the sample. Figure 5b–d shows the spectra of B 1s, C 1s, and N 1s. In the B 1s XPS spectrum (Figure 5b), two peaks centered at 191.3 and 192.4 eV are identified, which can be attributed to the B–C and B–N bonding structures [20]. The C 1s signal (Figure 5c) could be ascribed to four peaks at approximately 284.5, 285.1, 286.6, and 288.5 eV, corresponding to the C–B, C–C, C–N, and C–O bonds, respectively [21]. The high-resolution N 1s spectrum (Figure 5d) can be deconvoluted into three peaks at 398.7, 399.8, 401.2 eV, which are assigned to the N–B, graphitic N–C, and N=C bonds, respectively [22].

**Figure 6:**
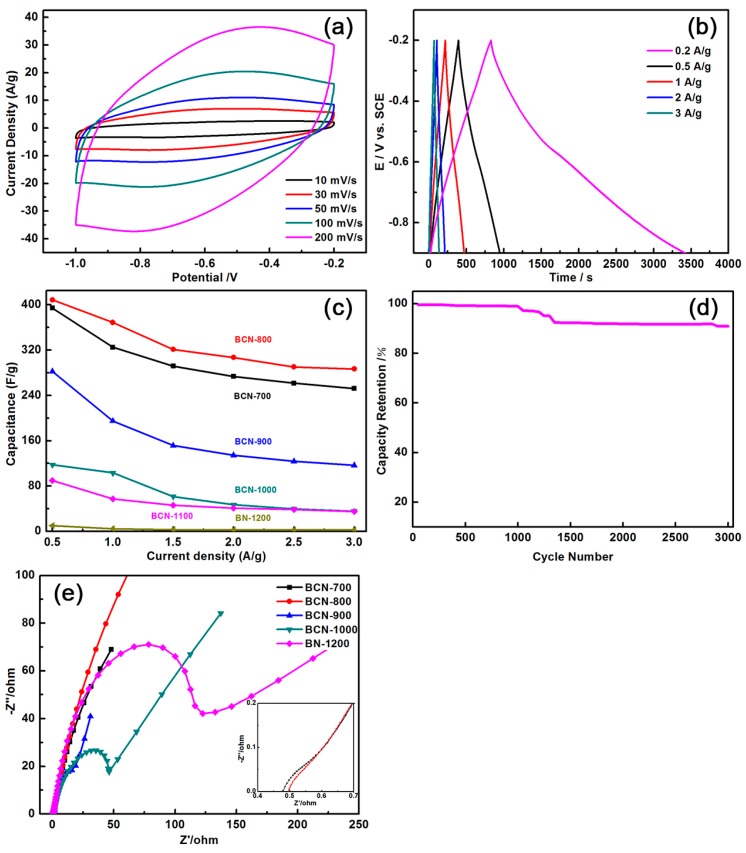
(**a**) Cyclic voltammetry (CV) curves of BCN-800 at various scan rates in 6.0 M KOH electrolyte solution; (**b**) Galvanostatic charge/discharge curves of BCN-800 at a current density of 0.2 A/g in 6.0 M KOH electrolyte solution; (**c**) The specific capacitance at the current density from 0.5 to 3 A/g; (**d**) the stability evaluation of BCN-800 electrode material in 6 M KOH solution at a charge current of 8 A/g; (**e**) Nyquist plots of samples in 6 M KOH solution. The inset shows the enlarged Nyquist plots in the high-frequency area of BCN-700 and BCN-800.

Cyclic voltammetry (CV) was employed with 6.0 M KOH as electrolyte in a three-electrode system and a potential interval between −1 V and −0.2 V to investigate the electrochemical behavior of the as-prepared samples. Figure 6a shows the CV curves of the BCN-800 electrode at different scan rates of 10, 30, 50, 100, and 200 mV/s in 6.0 M KOH solution. As illustrated in Figure 6a, the curves exhibited a typical rectangular-like shape without a redox peak existing in the sample, which indicated the capacitive response from the electrical double-layer capacitor. The comparison of the curves shows that the enclosed area of the CV curve decreased drastically with the increase in temperature, which indicates that lowering the reaction temperature might enhance the curve area (as seen in the Supplementary Figure S9(a)).

Furthermore, galvanostatic charge/discharge experiments were performed to calculate the specific capacitance. Figure 6b shows the galvanostatic charge/discharge of BCN-800 with different current densities in 6.0 M KOH solution. According to the formula *It*/*E* (where *t* is the discharge time, *I* is the charge/discharge current, and *E* is the voltage difference), the specific capacitances of BCN-700, BCN-800, BCN-900, BCN-1000, BCN-1100, and BN-1200 were 615.5 F/g, 805.9 F/g, 288.3 F/g, 221.6 F/g, 173.2 F/g, 14.4 F/g at a current density of 0.2 A/g, respectively. Among them, BCN-800 showed the largest specific capacitance. At the same current density, the specific capacitance of BCN-800 was significantly higher than that of VA-BC_2_NNTAs (547 F/g) and VA-BCN (321 F/g) [23,24,25]. The primary reason for this result was influenced by the morphology. Previous SEM and TEM tests showed that the morphology of BCN-800 was “ravine-like”, which increases the contact area between electrode and electrolyte, thereby increasing the ion exchange between electrode and electrolyte and improving the specific capacitance of the BCN-800 electrode.

For an ideal supercapacitor, it is important to explore whether it can guarantee the same energy and good cycle stability under different operating conditions. Thus, this study investigated the capacitance retention rates of different samples. Figure 6c shows the specific capacitance obtained at current densities of 0.5, 1, 1.5, 2, 2.5, and 3 A/g to illustrate the rate capability. For the BCN-800 electrode, when the discharge current increased from 0.5 A/g to 3 A/g, the specific capacitance could still be maintained at 70.18%. However, for the BCN-700, BCN-900, BCN-1000, BCN-1100, and BN-1200 electrodes, the capacitance retention rates were only approximately 64.00%, 68.00%, 45.75%, 39.78%, and 24.51%, respectively. The durability of the BCN-800 electrode was examined by the continuous charge/discharge test at a current density of 8 A/g. Figure 6d shows that capacitance could still be maintained at 91% after 3000 cycles, which indicates that the BCN-800 electrode had good stability and high rate capability.

Electrochemical impedance spectroscopy (EIS) was studied for samples. The Nyquist plots are shown in Figure 6e, the semicircles of BCN-800 and BCN-700 are both smaller in the high-frequency range, indicating that both samples had smaller charge transfer resistance. However, the charge transfer resistance of BCN-700 was greater than that of BCN-800 (inset). With the increase of pyrolysis temperature, the semicircle of samples in the high-frequency range showed a significant increase of charge transfer resistance. The BCN-800 had the largest straight-line slope in the low-frequency region, indicating that it had the smallest electrolyte diffusion resistance and the best capacitance behavior. From the EIS studies, it can be concluded that BCN-800 showed significant capacitive behavior and lower electrochemical charge transfer resistance. This conclusion is consistent with the results of electrochemical specific capacitance test.

## 3. Materials and Methods

### 3.1. Preparation of the Precursor

The “ravine-like” BCN compounds were synthesized via precursor pyrolysis. Firstly, boric acid (0.886 g) was thoroughly mixed with 2,4,6-tri(2-pyridyl)-1,3,5-triazine (0.5 g). Then, the mixture was dissolved in 20 mL of acetonitrile. The solution was kept in a 250 mL round-bottom flask with stirring, followed by refluxing at 70 °C for 8 h. The precursor was collected after vacuum filtration and further drying overnight at 80 °C.

### 3.2. Synthesis of “Ravine-Like” BCN Compounds

The as-prepared precursor was placed in a corundum boat. Then, the boat was placed in a tube furnace and subsequently purged under nitrogen. The constant nitrogen flow was maintained throughout the entire procedure to obtain an inert atmosphere. The boat was held at 600 °C, 700 °C, 800 °C, 900 °C, 1000 °C, 1100 °C, and 1200 °C for 3 h with a heating rate of 10 °C/min, respectively. The final products (named BCN-temperature) were obtained after washing with deionized water and drying overnight.

### 3.3. Characterization

Fourier transform infrared (FTIR) spectroscopy was conducted using the Nicolet iS50 to characterize the chemical structure. X-ray diffraction (XRD) patterns were observed using the Rigaku MiniFlex 600 (SCINCO CHINA, Shanghai, China) with Cu-Kα radiation to analyze the crystal structure. Scanning electron microscopy (SEM) images were obtained using the Hitachi field emission scanning electron microscope, and transmission electron microscopy (TEM) images were collected through the JEM-2100 to examine the morphology and structure of the samples. X-ray photoelectron spectroscopy (XPS) was conducted using the Thermo ESCALAB 250 (Thermo Fisher Scientific, Shanghai, China) with an X-ray Al Kα source to analyze the bonding state.

## 4. Conclusions

In summary, a facile synthesis method was presented to fabricate a series of “ravine-like” ternary BCN compounds. The method has the characteristics of brief reaction steps, high yield, and low cost. The range for synthesized BCN compounds was 700 to 1100 °C. The BCN-800 electrode exhibited the highest specific capacitance, which is 805.9 F/g at a current density of 0.2 A/g. This finding can be attributed to the “ravine-like” morphology of BCN-800 that increases the contact area between electrode and electrolyte and facilitates ion exchange. In addition, the BCN-800 electrode presented excellent rate capability and good cycling stability (91%) after 3000 cycles at a current density of 8 A/g. This excellent performance suggests high potential applications of BCN material in supercapacitors.

## Figures and Tables

**Table 1:** Quantitative elemental compositions of the samples derived from XPS surveys.

Sample	B (at %)	C (at %)	N (at %)	O (at %)
BCN-700	13.02	52.54	13.83	20.61
BCN-800	10.15	62.6	12.17	15.02
BCN-900	20.94	48.7	16.23	14.11
BCN-1000	43.09	14.0	32.9	9.97
BCN-1100	40.87	14.9	30.9	13.1
BN-1200	49.05	4.37	39.5	7.01

## Supplementary Materials

The following are available online at http://www.mdpi.com/1996-1944/11/2/209/s1, Figure S1: TEM images and the corresponding HRTEM images of BCN-1000; Figure S1A: TEM images and the corresponding HRTEM images of BCN-1100; Figure S1B: TEM images and the corresponding HRTEM images of BN-1200; Figure S2: EDS and Elemental Mapping of BCN-800; Figure S2A: EDS and Elemental Mapping of BCN-1000; Figure S2B. EDS and Elemental Mapping of BN-1200; Figure S3. Adsorption and desorption curve of samples; (a) BCN-800; (b) BCN-900; (c) BCN-1000; (d) BCN-1100; (e) BN-1200; Figure S4. (a) The survey scan of XPS on BCN-700; (b) B 1s XPS peak; (c) C 1s XPS peak and (d) N 1s XPS peak; Figure S5. (a) The survey scan of XPS on BCN-900; (b) B 1s XPS peak; (c) C 1s XPS peak and (d) N 1s XPS peak; Figure S6. (a) The survey scan of XPS on BCN-1000; (b) B 1s XPS peak; (c) C 1s XPS peak and (d) N 1s XPS peak; Figure S7. (a) The survey scan of XPS on BCN-1100; (b) B 1s XPS peak; (c) C 1s XPS peak and (d) N 1s XPS peak; Figure S8. (a) The survey scan of XPS on BN-1200; (b) B 1s XPS peak; (c) C 1s XPS peak and (d) N 1s XPS peak; Figure S9. (a) CV curves of BCN-800, BCN-900, BCN-1000, BCN-1100 and BN-1200 at a scan rate of 50 mV/s; (b) discharge curves of samples obtained at different pyrolysis temperatures; Figure S10. CV curves of BCN-700 at various scan rates in 6.0 M KOH electrolyte solution; Table S1: Specific surface area of samples.
