# Supplementary material for: Synthesis and Characterization of “Ravine-Like” BCN Compounds with High Capacitance
Source: Materials (Basel). 2018 Jan 29;11(2):209. doi: 10.3390/ma11020209 (PMC5848906; doi:10.3390/ma11020209)

Supporting Information

Synthesis and Characterization of "Ravine-like" BCN Compounds with High Capacitance

Dongping Chen, Yanzhen Huang, Xinling Hu, Rongkai Li, Yingjiang Qian and Dongxu Li*

College of Materials Science and Engineering, Huaqiao University, Xiamen 361021, P. R. China

E-mail: lidongxu@hqu.edu.cn

Contents

Figure S1	TEM images and the corresponding HRTEM images of BCN-1000	S2
Figure S1A	TEM images and the corresponding HRTEM images of BCN-1100	S2
Figure S1B	TEM images and the corresponding HRTEM images of BN-1200	S3
Figure S2	EDS and Elemental Mapping of BCN-800	S3
Figure S2A	EDS and Elemental Mapping of BCN-1000	S4
Figure S2B	EDS and Elemental Mapping of BN-1200	S5
Figure S3	Adsorption and desorption curve of samples; (a) BCN-800; (b) BCN-900; (c) BCN-1000; (d) BCN-1100; (e) BN-1200	S6
Table S1	Specific surface area of samples	S6
Figure S4	(a) The survey scan of XPS on BCN-700; (b) B 1s XPS peak; (c) C 1s XPS peak and (d) N 1s XPS peak	S7
Figure S5	(a) The survey scan of XPS on BCN-900; (b) B 1s XPS peak; (c) C 1s XPS peak and (d) N 1s XPS peak	S8
Figure S6	(a) The survey scan of XPS on BCN-1000; (b) B 1s XPS peak; (c) C 1s XPS peak and (d) N 1s XPS peak	S9
Figure S7	(a) The survey scan of XPS on BCN-1100; (b) B 1s XPS peak; (c) C 1s XPS peak and (d) N 1s XPS peak	S10

Figure S8	(a) The survey scan of XPS on BN-1200; (b) B 1s XPS peak; (c) C 1s XPS peak and (d) N 1s XPS peak	S11
Figure S9	(a) CV curves of BCN-800, BCN-900, BCN-1000, BCN-1100, BN-1200 at a scan rate of 50 mV/s; (b) discharge curves of samples obtained at different pyrolysis temperatures	S12
Figure S10	CV curves of BCN-700 at various scan rates in 6.0 M KOH electrolyte solution	S12

Figure S1. TEM images and the corresponding HRTEM images of BCN-1000

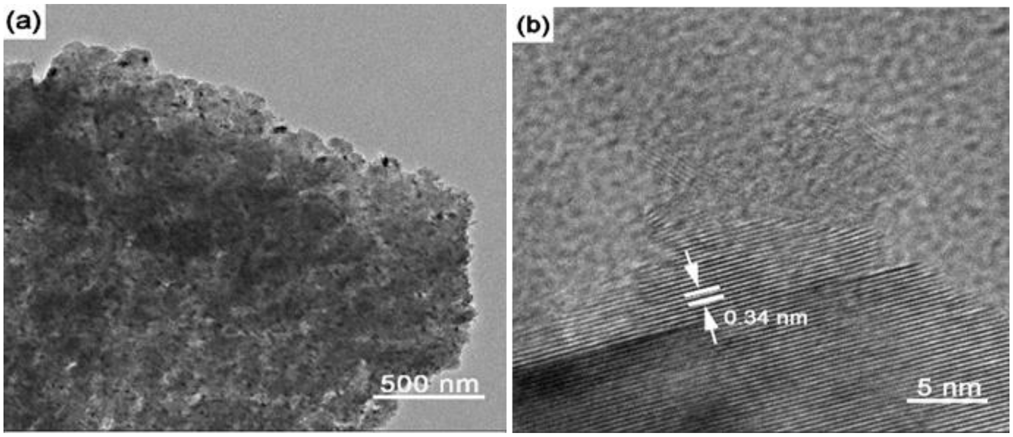

Figure S1A. TEM images and the corresponding HRTEM images of BCN-1100

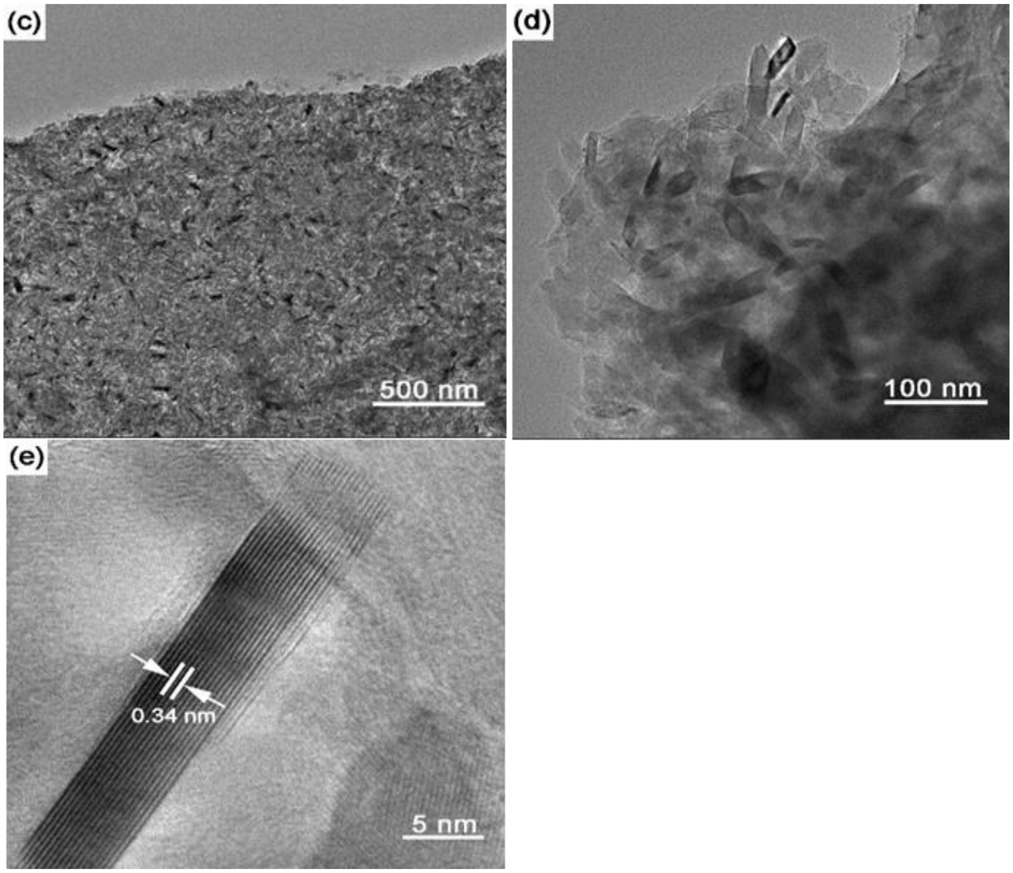

Figure S1B. TEM images and the corresponding HRTEM images of BN-1200

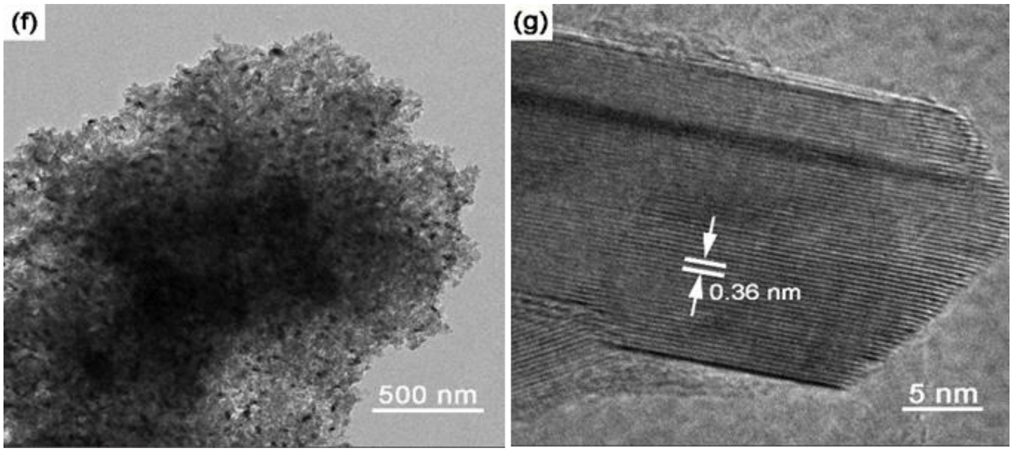

Figure S2. EDS and Elemental Mapping of BCN-800

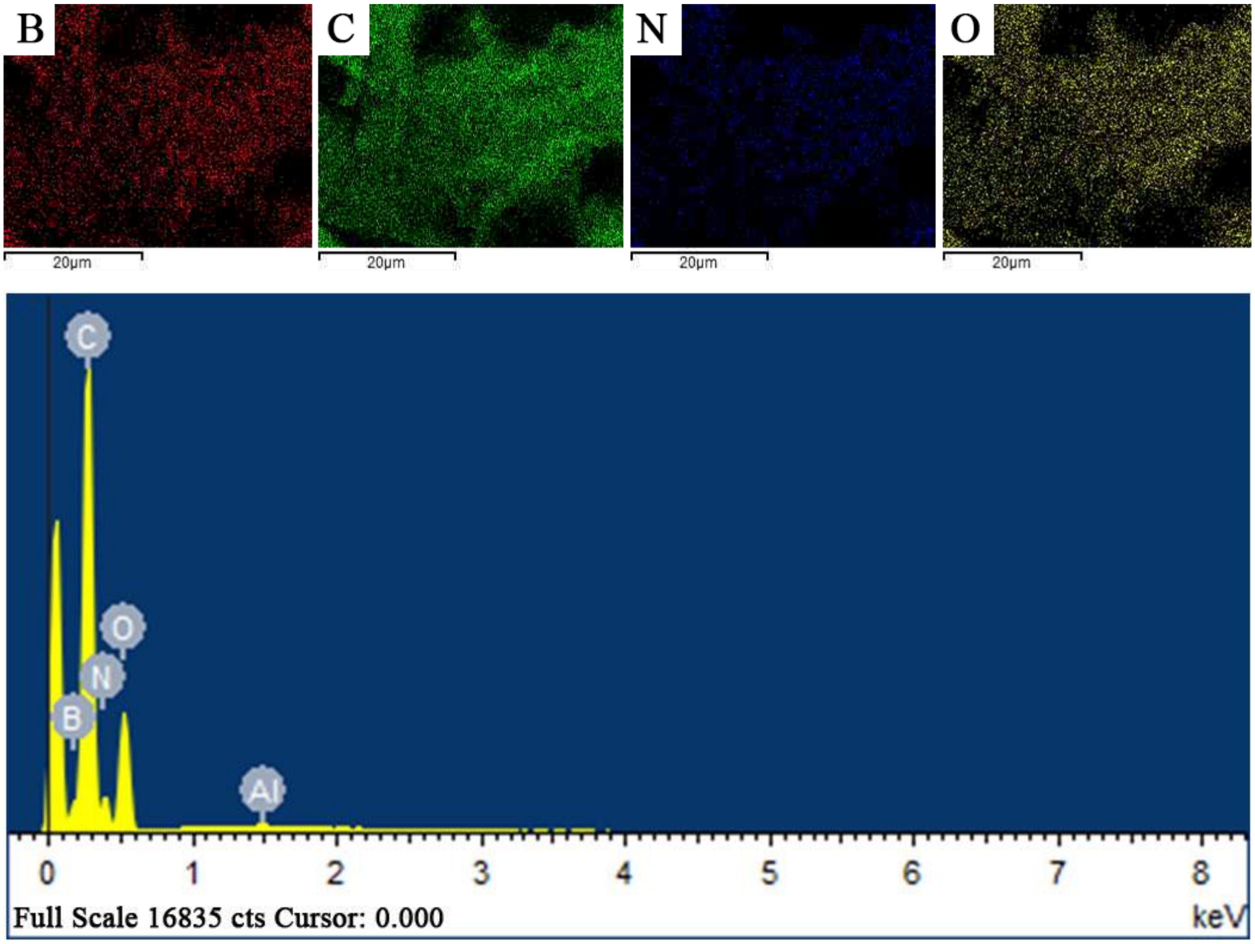

Figure S2A. EDS and Elemental Mapping of BCN-1000

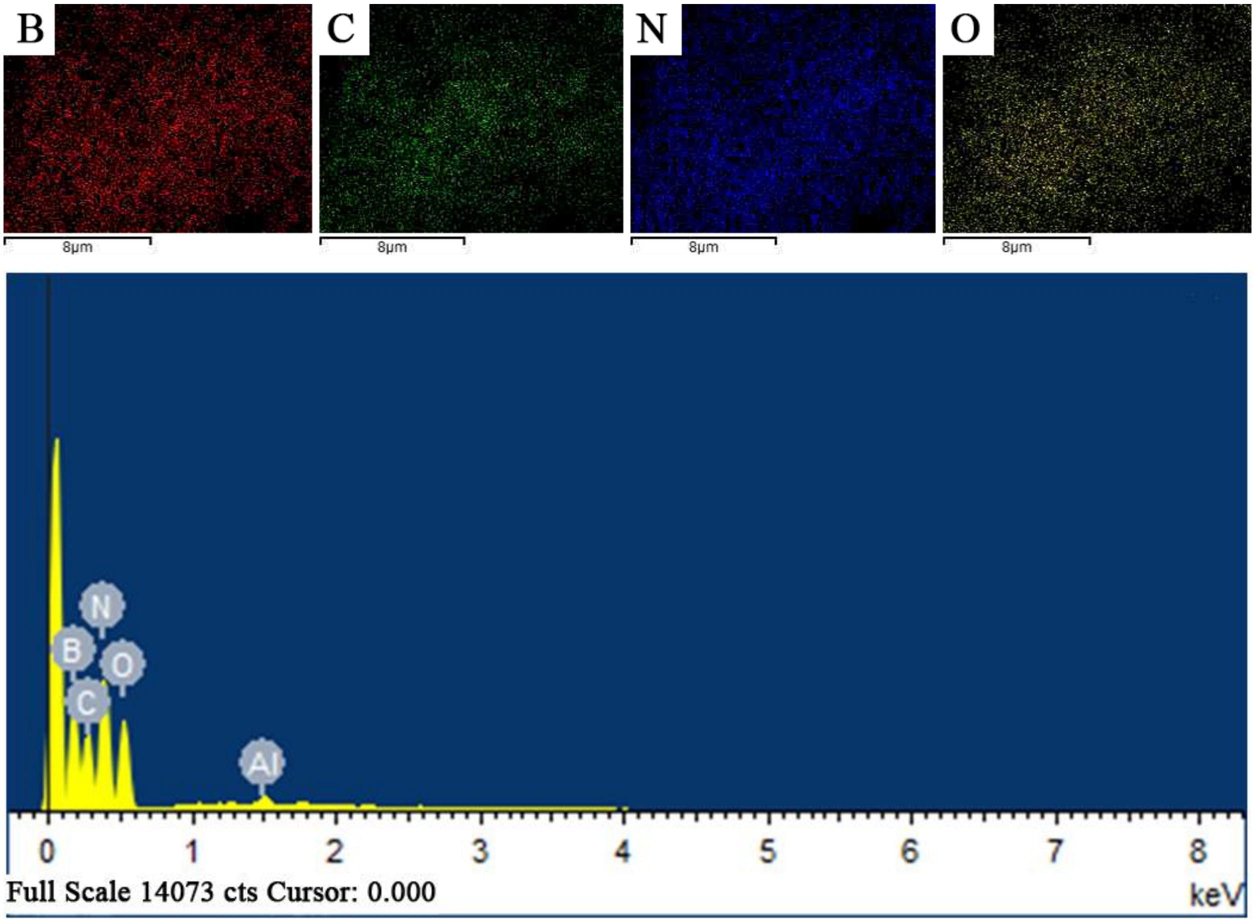

Figure S2B. EDS and Elemental Mapping of BN-1200

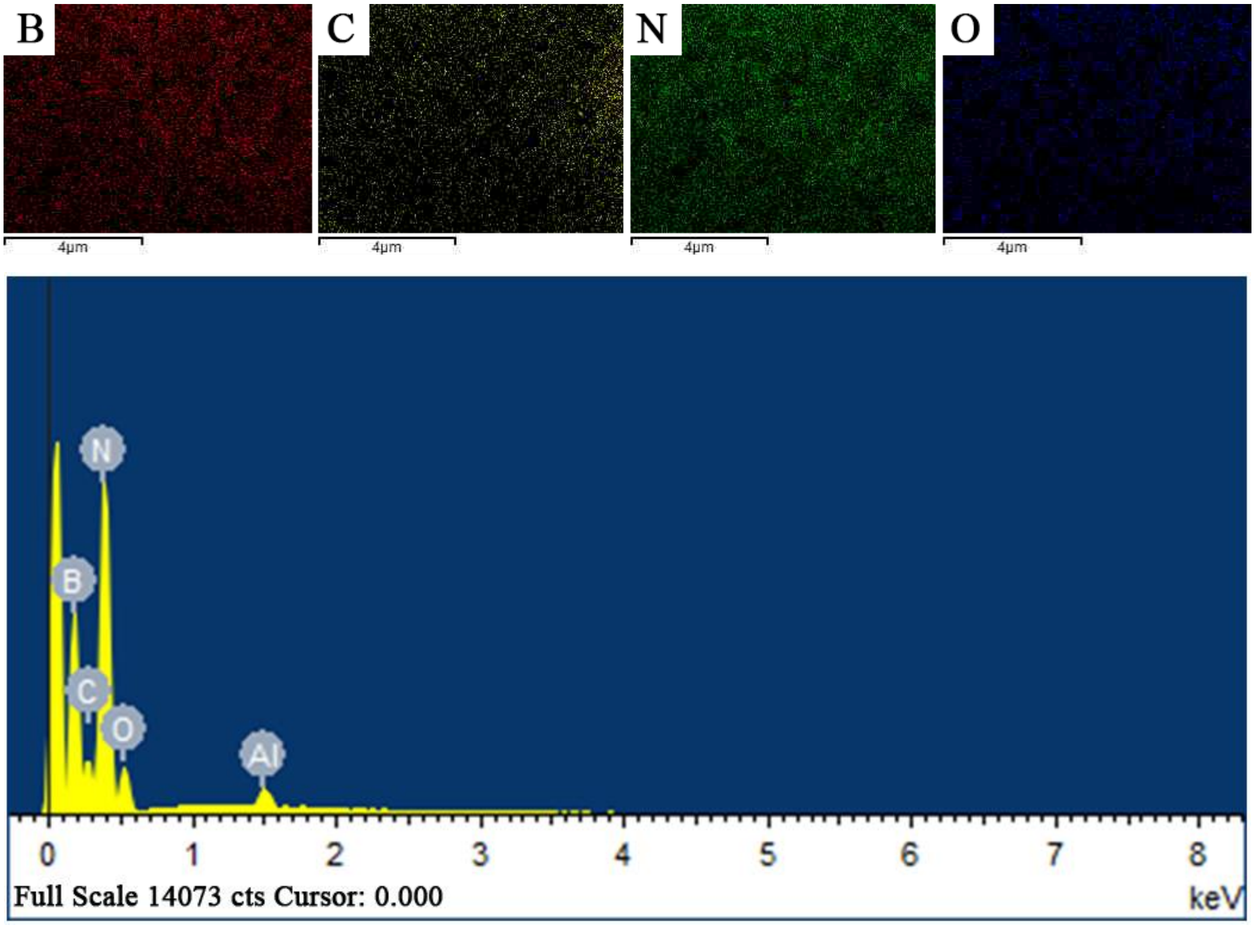

Figure S3. Adsorption and desorption curve of samples; (a) BCN-800; (b) BCN-900; (c) BCN-1000; (d) BCN-1100; (e) BN-1200

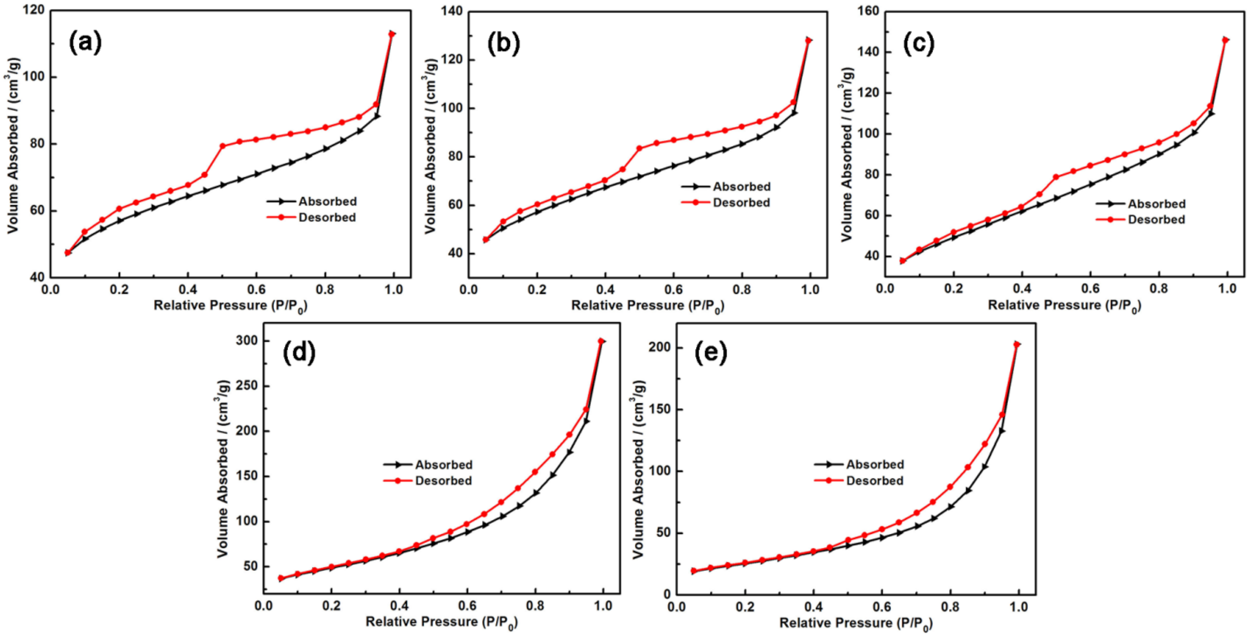

Table S1. Specific surface area of samples

Sample	BCN-800	BCN-900	BCN-1000	BCN-1100	BN-1200
**specific surface area**	198.0 m^2/g	193.4 m^2/g	174.9 m^2/g	175.9 m^2/g	92.9 m^2/g

Figure S4. (a) The survey scan of XPS on BCN-700; (b) B 1s XPS peak; (c) C 1s XPS peak and (d) N 1s XPS peak.

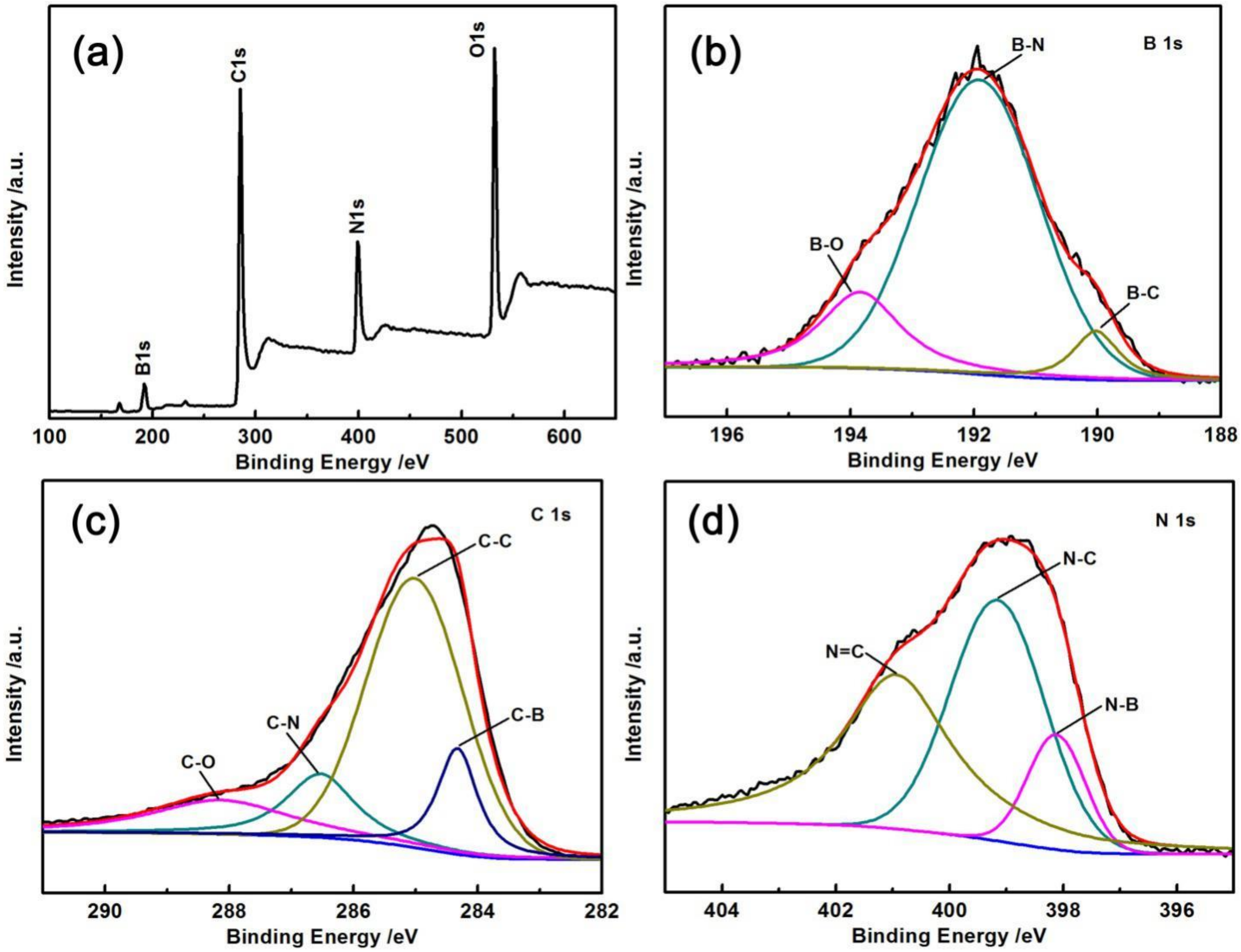

Figure S5. (a) The survey scan of XPS on BCN-900; (b) B 1s XPS peak; (c) C 1s XPS peak and (d) N 1s XPS peak

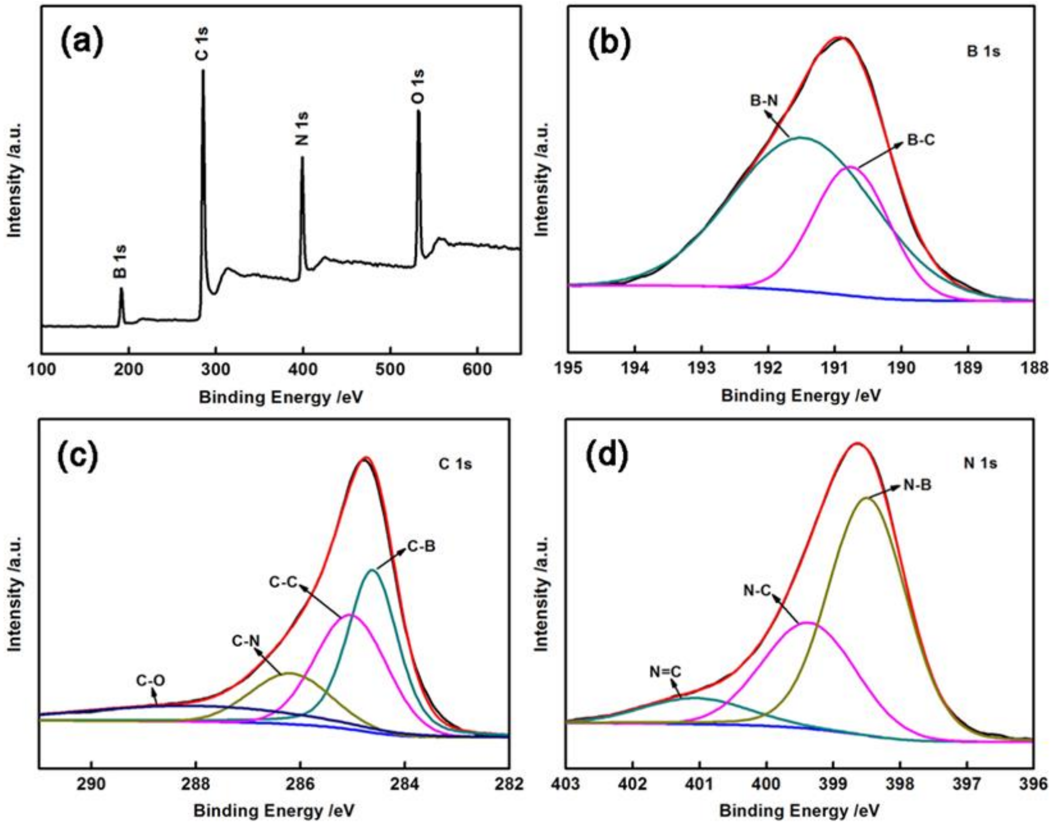

Figure S6. (a) The survey scan of XPS on BCN-1000; (b) B 1s XPS peak; (c) C 1s XPS peak and (d) N 1s XPS peak

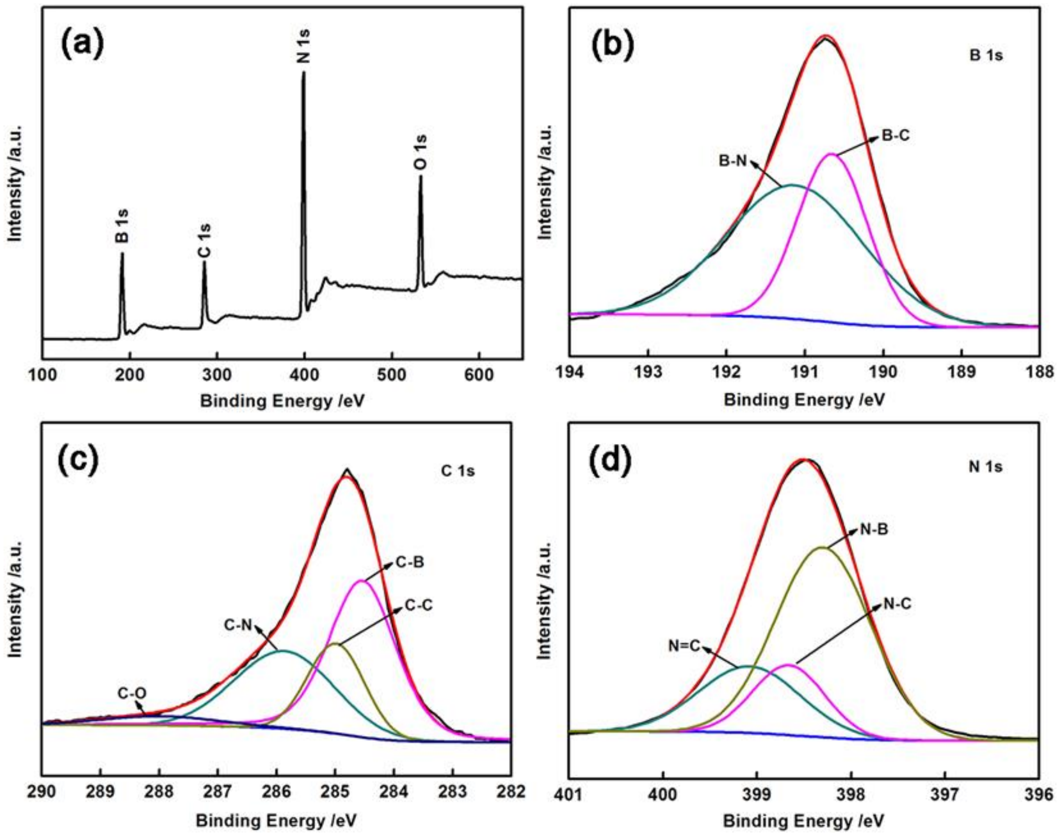

Figure S7. (a) The survey scan of XPS on BCN-1100; (b) B 1s XPS peak; (c) C 1s XPS peak and (d) N 1s XPS peak

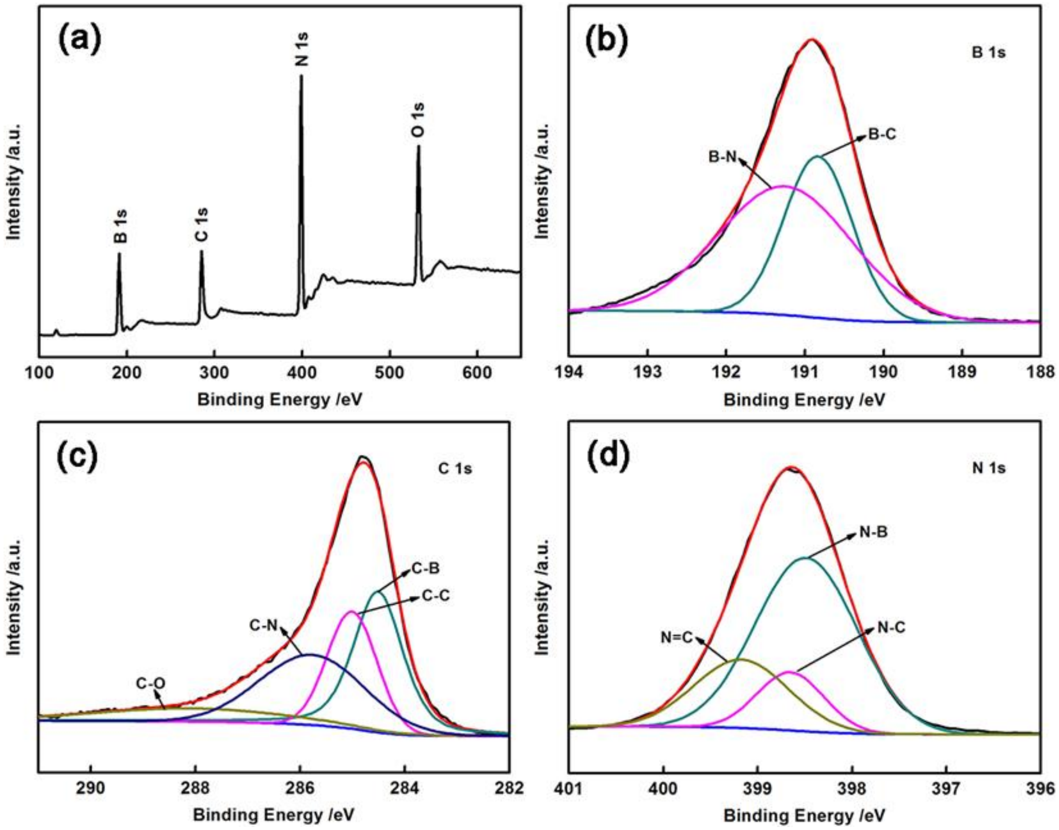

Figure S8. (a) The survey scan of XPS on BN-1200; (b) B 1s XPS peak; (c) C 1s XPS peak and (d) N 1s XPS peak

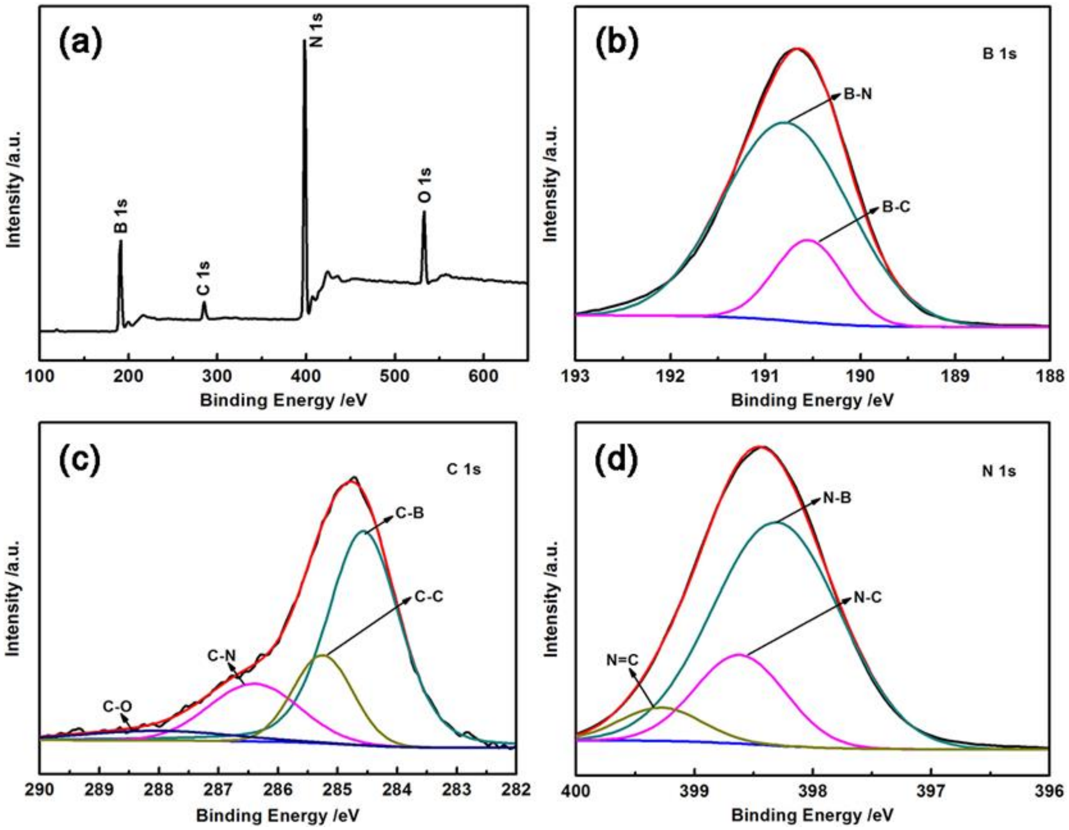

Preparation of electrode.

The active materials (i.e., BCN-700, BCN-800, BCN-900, BCN-1000, BCN-1100, and BN-1200), acetylene black and poly tetra fluoro ethylene (PTFE) binder are mixed in mass ratio of 8: 1: 1. The mixture was dissolved in absolute ethyl alcohol and ultrasound to form homogenous slurries. The homogenous slurries were coated onto cleaned nickel mesh (1 cm^2 area, 5% hydrochloric acid, ethanol and deionized water each ultrasonic cleaning 20 min) and further dried at 60 °C for 12 h under vacuum. As-formed electrodes were then pressed at a pressure of 4 MPa and the loading in final electrodes is 2.8 mg.

Figure S9. (a) CV curves of BCN-800, BCN-900, BCN-1000, BCN-1100 and BN-1200 at a scan rate of 50 mV/s; (b) discharge curves of samples obtained at different pyrolysis temperatures

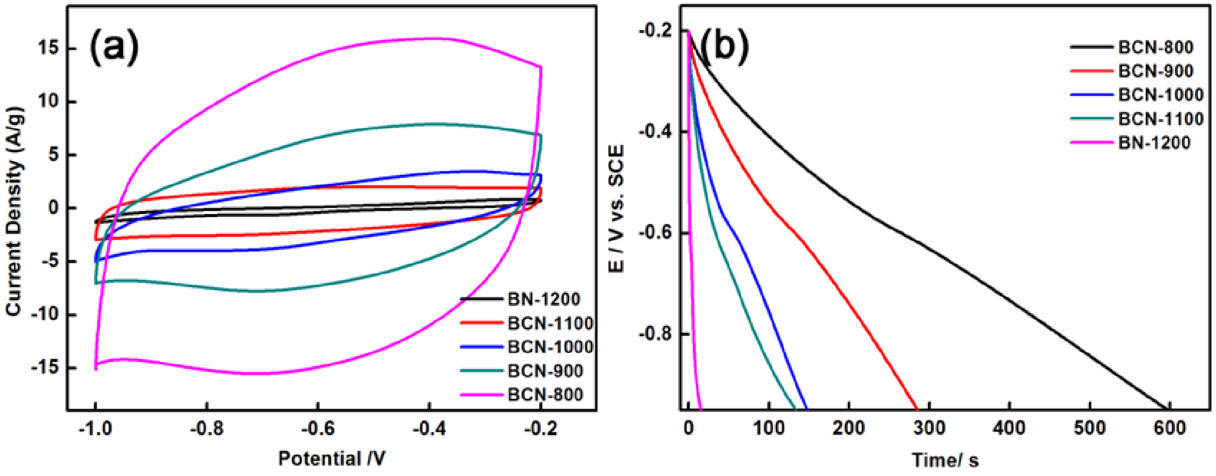

Figure S10. CV curves of BCN-700 at various scan rates in 6.0 M KOH electrolyte solution

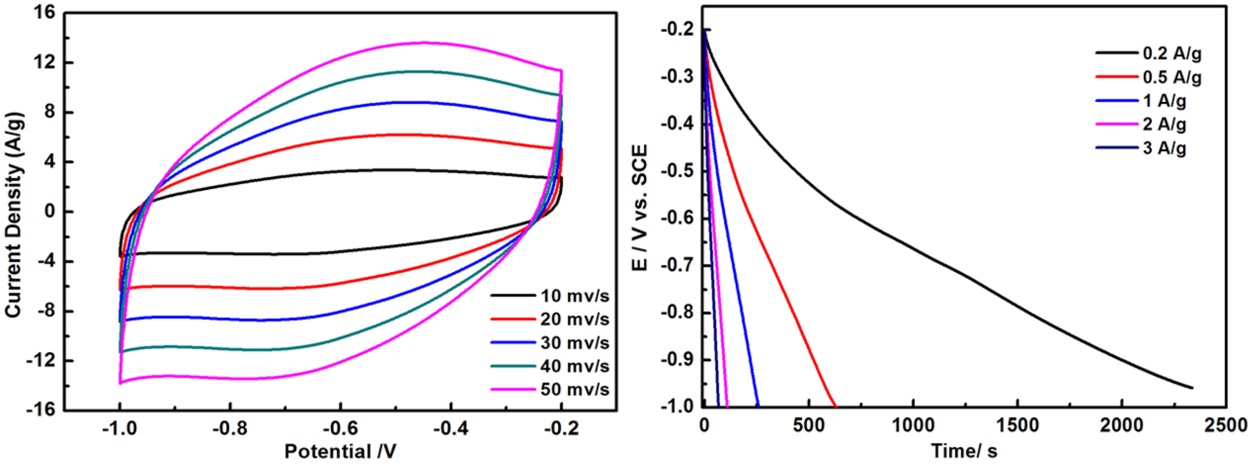

Supplement: Supplementary file 1 [file materials-11-00209-s001.pdf]
